# LncRNA Fendrr: involvement in the protective role of nucleolin against H_2_O_2_-induced injury in cardiomyocytes

**DOI:** 10.1080/13510002.2023.2168626

**Published:** 2023-01-31

**Authors:** Cheng Chen, Xiaofang Lin, Yuting Tang, Hui Sun, Leijing Yin, Zhengyang Luo, Shuxin Wang, Pengfei Liang, Bimei Jiang

**Affiliations:** a Department of Pathophysiology, Sepsis Translational Medicine Key Laboratory of Hunan Province, Xiangya School of Medicine, Central South University, Changsha, People’s Republic of China; b Department of Burns and Plastic Surgery, Xiangya Hospital, Central South University, Changsha, People’s Republic of China

**Keywords:** Nucleolin, lncRNA, Fendrr, hydrogen peroxide, oxidative stress, apoptosis, injury, cardiomyocyte

## Abstract

**Background:** Nucleolin is a multifunctional nucleolar protein with RNA-binding properties. Increased nucleolin expression protects cells from H_2_O_2_-induced damage, but the mechanism remains unknown. Long noncoding RNAs (lncRNAs) play crucial roles in cardiovascular diseases. However, the biological functions and underlying mechanisms of lncRNAs in myocardial injury remain unclear.

**Methods:** In a nucleolin-overexpressing cardiac cell line, high-throughput technology was used to identify lncRNAs controlled by nucleolin. Cell counting kit-8 assay was used to determine cell viability, lactate dehydrogenase (LDH) assay to detect cell death, caspase activity assay and propidium iodide staining to confirm cell apoptosis, and RNA immunoprecipitation to examine the interaction between Fendrr and nucleolin.

**Results:** We found that Fendrr expression was significantly downregulated in mouse hearts subjected to myocardial ischemia-reperfusion (MI/R) injury. High Fendrr expression abrogated H_2_O_2_-mediated injury in cardiomyocytes as evidenced by increased cell viability and decreased cell apoptosis. Conversely, Fendrr knockdown exacerbated the cardiomyocytes injury. Also, nucleolin overexpression inhibits Fendrr downregulation in H_2_O_2_-induced cardiomyocyte injury. Fendrr overexpression significantly reversed the role of the suppression of nucleolin expression in H_2_O_2_-induced cardiomyocytes.

**Conclusion:** LncRNA Fendrr is involved in the cardioprotective effect of nucleolin against H_2_O_2_-induced injury and may be a potential therapeutic target for oxidative stress-induced myocardial injury.

## Introduction

Nucleolin (also known as C23) is a multifunctional protein commonly expressed in eukaryotic cells [1]. Nucleolin is essential for ribosome biogenesis and RNA metabolism [2]. It participates in a variety of biological processes, including gene replication, transcription, gene silencing, cell cycle regulation, and cell death [3]. Our previous research has shown that oxidative stress causes nucleolin cleavage and apoptosis in C2C12 myogenic [4] and human umbilical vein endothelial cells [5]. Nucleolin overexpression inhibits hydrogen peroxide (H_2_O_2_)-induced apoptosis [6]. It protects the heart by interacting with other molecules, resulting in a smaller infarcted area and reduced cardiomyocyte death [7, 8]. Although research has shown that nucleolin plays a significant protective role in cardiomyocyte injury, its precise molecular mechanism remains unknown.

The protein-coding genes account for only approximately 2–3% of the human genome, and the majority of the remaining genes are transcribed into noncoding RNA (ncRNA) [9]. Long ncRNAs (lncRNAs) are transcripts with more than 200 nucleotides and limited coding potential. The nomenclature of lncRNAs depends on multiple characteristics such as the transcription initiation point, cell or tissue specificity, molecular function, and mechanism of action [10]. Recently, lncRNAs have been implicated as important gene regulators in cardiac remodeling, myocardial hypertrophy, and myocardial ischemia-reperfusion (MI/R) injury [11–13]. The rapid development of RNA sequencing technology has revealed that lncRNAs are abnormally expressed in various diseases. The biological functions and mechanisms of lncRNAs in various diseases have also been extensively studied.

Cardiovascular diseases and their consequences are the most common health problems in humans. Extensive research has been conducted on the pathogenesis of cardiovascular disease, such as oxidative stress and activation of cell death systems [14]. In vitro H_2_O_2_ stimulation of cardiomyocytes is the most common way to simulate MI/R models [15–17]. Recently, new molecular mechanisms have been proposed for cardioprotection against MI/R injury. However, effective drugs and methods for improving reperfusion-related injuries are still lacking. Therefore, an in-depth understanding of the mechanism underlying reperfusion injury and identification of new targets are crucial for preventing and treating cardiomyocyte injury.

This study aimed to investigate the molecular mechanism underlying the protective effect of nucleolin against cardiomyocyte injury. The expression profile of lncRNAs in cardiomyocytes overexpressing nucleolin was elucidated using high-throughput screening technology. We used a mouse model of MI/R injury to detect the expression of lncRNA Fendrr and observed the protective effect of Fendrr in H_2_O_2_-induced cardiomyocyte injury. We found that Fendrr is relevant to the protective role of nucleolin, which regulates Fendrr expression. This suggests lncRNA Fendrr involvement in the cardioprotective effect exerted by nucleolin against H_2_O_2_-induced injury.

## Material and methods

### Animal experiments

All animal experiments were performed in conformance with the Animal Experimentation Guidelines of the Medical Ethics Committee of Xiangya Hospital and Central South University (No. 201402027). The animals were subjected to cardiac ischemia-reperfusion damage as previously described (Jiang et al., 2013, 2014). Briefly, BALB/c mice were separated into experimental (n = 3) and sham operation (n = 3) groups. The mice were sedated with pentobarbital (50 mg/kg) every 2 h. Under sterile conditions, left thoracotomy was performed in the fourth intercostal space to expose the heart. MI/R was induced by occluding the left anterior descending coronary artery (LAD) for 30 min, followed by reperfusion for 0, 2, 6, 12, and 24 h. The animals in the sham group underwent the same procedures as those in the experimental group with the exception of ligation. Following reperfusion, the heart was removed at specific time points for histological and morphological examination.

### LncRNA profiling

Using the Aglient Rat lncRNA microarray V2.0 (Arraystar, Rockville, MD, USA), three sample pairs were prepared for lncRNA microarray analysis in the nucleolin-overexpressing rat cardiomyocyte cell line and control group. The slides were incubated for 17 h in an Agilent hybridization chamber at 65°C and scanned using the Agilent scanner G2505B. Agilent Feature Extraction software (version 11.0.1.1) was used to analyze the acquired array images. Quantile normalization and subsequent data processing were conducted using GeneSpring GX v12.1 software package (Agilent Technologies, Santa Clara, CA, USA). Differentially expressed lncRNAs between the two groups were identified through paired t-test (*P* < 0.05 and fold-change > 2.0). All microarray experiments were performed by Kangcheng Bio-Tec (Shanghai, China).

### Immunofluorescence assays and fluorescence in situ hybridization (FISH)

Cy3-labeled Fendrr and a Fluorescent *In Situ* Hybridization Kit (RiboBio, Guangzhou, China) were used according to the manufacturer's instructions. Briefly, H9c2 cells were fixed with 4% v/v paraformaldehyde for 10-min after the cells attained 70–80% confluency. Hybridization was then performed at 37°C overnight using a Fendrr probe. Subsequently, the cells were stained with DAPI and the images were acquired using an Olympus IX71 inverted microscope.

### Cell culture and treatment

H9c2 cells were obtained from the American Type Culture Collection and cultured in Dulbecco's modified Eagle's medium supplemented with 10% v/v fetal bovine serum. The cells were cultured at 37 °C in a humidified incubator under 5% v/v CO_2_. At 70–80% confluence, cellular oxidative stress was induced by exposure to different H_2_O_2_ concentrations for different time periods.

### Cell transfection experiments

After the H9c2 cells attained 70–80% confluency, they were transfected with recombinant lentiviruses housing the pHS-AVC-LW1120 (Fendrr) and pHS-BVC-LW280 (negative control) constructs (Syngentech; Beijing, China) for 72 h. Also, the H9c2 cells at 80% confluency were transfected with a nucleolin plasmid (pcDNA3-Nuc) and incubated in a CO_2_ incubator for 48 h at 37°C. For small interfering RNA (siRNA) transfection, nucleolin or Fendrr siRNA (si-C23 or si-Fendrr) and the corresponding negative control siRNA (si-NC or si-nc; Invitrogen Life Technologies) were transfected into cells. Lipofectamine 2000 reagent (Invitrogen) was used for cell transfection according to the manufacturer's instructions. After 48 h of transfection, the cells were lysed for further experiments.

### Propidium iodide staining

Propidium iodide (PI) fluorescence staining was used to detect cell death. In summary, the cells were stained with 1 µg/mL of PI staining reagent (Dingguo Biotechnology Co., Ltd.) for 10 min, washed twice with PBS, and observed. The cells positive for apoptosis displayed red fluorescence. Honchest was used for nuclear staining.

### RNA isolation and qRT-PCR analysis

Total RNA was isolated from mouse myocardial tissues and cardiomyocytes using TRIzol reagent (Invitrogen, Carlsbad, CA, USA) according to the manufacturer's instructions. Total RNA was reverse-transcribed into complementary DNA using a Reverse Transcription Kit (Takara, Dalian, China). To determine Fendrr expression levels, quantitative reverse transcription polymerase chain reaction (RT-qPCR) was performed using an Applied Biosystems 7500 Fast Real-Time PCR System (Foster City, CA, USA). GADPH was used as an internal control in myocardial tissues and cardiomyocytes. Relative gene expression levels were analyzed using the 2^−ΔΔCT^ method. The sequences of primers are shown in below table, primers analyses by NCBI blast search were used to identify the specificity of the amplified product.

**Table:** 

Gene	Accession number	Sequence
GAPDH (Mouse)	AY618199.1	Forward 5′- GGTGAAGGTCGGTGTGAACG- 3′
		Reverse 5′- CTCGCTCCTGGAAGATGGTG-3′
GAPDH (Rat)	NM_017008.4	Forward 5′- ACAGCAACAGGGTGGTGGAC-3′
		Reverse 5′- TTTGAGGGTGCAGCGAACTT-3′
Fendrr (Mouse)	NR_045471.2	Forward 5′- GGATGAAGACAGCCACAGTGACA-3′
		Reverse 5′- GGTCCACAAGATCCTTGGATAGAG-3′
Fendrr (Rat)	NR_126575.1	Forward 5′- TGTTGGCAGTACCATCTCGTCATG-3′
		Reverse 5′-GCATCCACAGTCAGGAAGCAGAG- 3′

### Cell viability assay

Cell viability was assessed using a Cell Counting Kit-8 assay (CCK-8; Dojindo, Kumamoto, Japan). The cultured cells were seeded at a density of 2-4 × 10^3^ cells/well in 96-well plates. After cell transfection, the medium was replaced with 100 μL of complete culture medium with or without H_2_O_2_. CCK-8 reagent (1:100) was added to the wells, and the wells were incubated for 1 h. The absorbance of the samples was measured at 450 nm using a microtiter reader. All experiments were performed in triplicates.

### Lactate dehydrogenase (LDH) assay

Cells were seeded in 6- or 12-well plates. After cell transfection, the medium was replaced with complete culture medium with or without H_2_O_2_. The assay was performed using a lactate dehydrogenase (LDH) assay kit (Nanjing Jiancheng Bioengineering Institute). Absorbance was measured using a microplate reader at a wavelength of 450 nm.

### Caspase activity assay

Caspase activity was analyzed using an ELISA kit (Abcam) according to the manufacturer’s instructions. Briefly, the cells were lysed using a homogenous reagent provided by the manufacturer. Lysates were then added to each well for analysis. Caspase-3 antibody (CST) was added with the detection reagent and incubated at room temperature for 15 min. The absorbance was measured at 450 nm.

### RNA immunoprecipitation (RIP) assay

The Magna RIP Kit (Millipore) was used for the RNA immunoprecipitation (RIP) assay according to the manufacturer's protocol. Cardiomyocytes were dissolved in RIP lysis buffer, followed by overnight incubation with anti-nucleolin antibody-conjugated protein A/G beads at 4 °C. After washing off the unbound materials, the RNAs bound to nucleolin were eluted and quantified. The purified RNA was analyzed using RT–PCR.

### Statistical analysis

The data were analyzed using SPSS 19.0 software. All data are presented as mean ± standard error of the mean (SEM). Differences between three or more groups were analyzed using Student's t-test and one-way analysis of variance (ANOVA). The results were considered statistically significant at *P *< 0.05.

## Results

### LncRNAs regulated by nucleolin in cardiomyocytes

**Figure 1.**
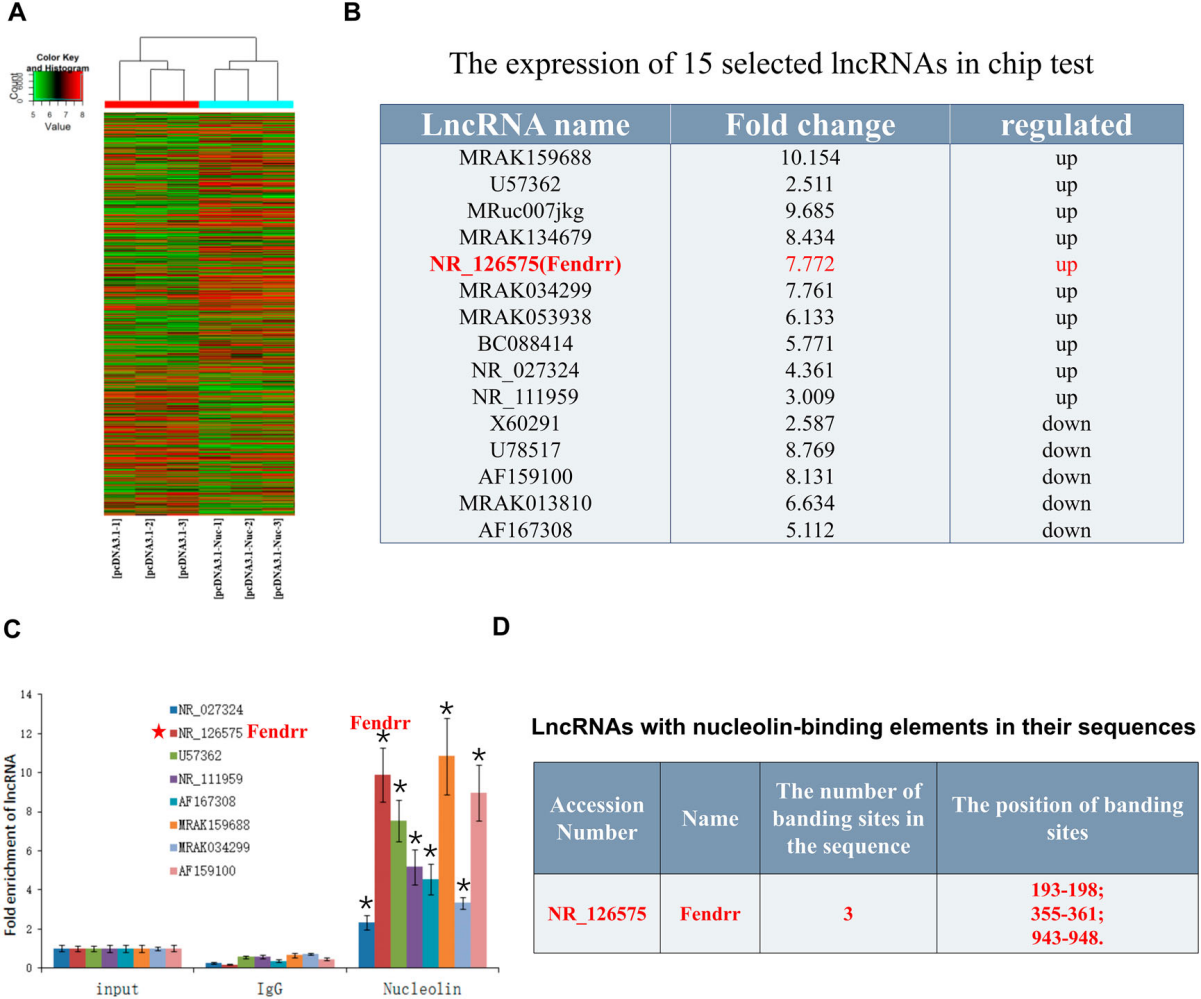
lncRNAs regulated by nucleolin in cardiomyocytes. (A) Heatmap profile of lncRNA microarray analysis. Green to red colors indicate low to high transcriptional levels. The lncRNAs differentially expressed between the two groups were identified through paired t-test *P* ≤ 0.05 and a fold change (FC) ≥ 2.5; n = 3 independent biological samples for each group. (B) The expression of 15 selected lncRNAs in chip test, where 10 differentially expressed up-regulated lncRNAs (up) and 5 differentially expressed down-regulated lncRNAs (down) were selected. (C) The interactions between nucleolin and lncRNAs were confirmed by RIP and identified via qRT-PCR. *, *P *< 0.05, vs. IgG group, n = 3. pcDNA3.1, the empty vector served as negative control; pcDNA3.1-Nuc, overexpression nucleolin group. (D) Bioinformatics website predicted Fendrr binding elements with nucleolin.

Nucleolin exerts a protective effect on the myocardium; however, the underlying mechanism is unclear. We used nucleolin-overexpressing cardiomyocyte cell lines to investigate the mechanism underlying the protective effect of nucleolin in the myocardium. In total, 267 differentially expressed lncRNAs (181 upregulated and 96 downregulated) were screened using high-throughput sequencing technology and compared with that in the control group (Figure 1A). These results suggest that nucleolin may regulate the expression of these lncRNAs. Based on the literature and chip data, we chose 15 differentially expressed lncRNAs (Figure 1B) for protein-RNA immunoprecipitation experiments. We discovered that eight lncRNAs including NR_027324, NR_126575 (Fendrr), U57362, NR_111959, AF167308, and MRAK159688 could bind to nucleolin (Figure 1C). Fendrr has multiple binding sites (Figure 1D), implying that nucleolin may directly bind and regulate Fendrr expression. However, few studies have explored the effects of Fendrr on myocardial injury, and in-depth research on Fendrr functional mechanisms in myocardial injury remains unexplored.

### LncRNA Fendrr expression down-regulated in myocardial injury

**Figure 2.**
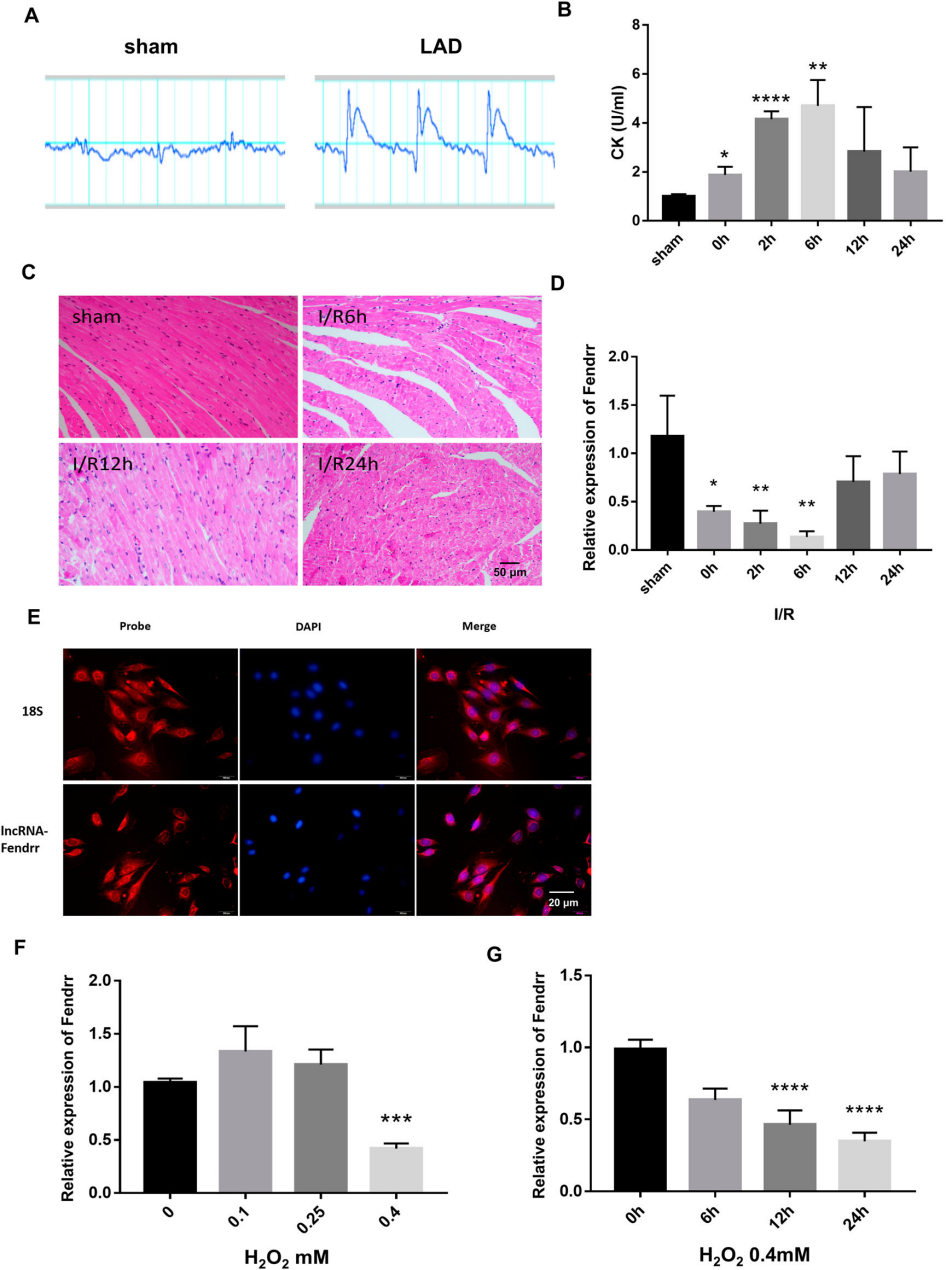
lncRNA Fendrr expression down-regulated in myocardial injury. (A) ECGs were recorded after ligating the left anterior descending coronary artery (LAD) in the mice hearts. Sham, sham operated control group; MI, myocardial ischemia group. (B) Serum creatine kinase (CK) value was detected after 30 min of ischemia followed by reperfusion for different time periods (0, 2, 6, 12, and 24 h) in mice (n = 3). Data have been represented as mean ± SD. *, *P*<0.05, vs. sham group, n = 3; **, *P*<0.01, n = 3, vs. sham group, n = 3; ****, *P*<0.0001, vs. sham group n = 3. (C) The representative hematoxylin-eosin staining images of the mice hearts after ischemia for 30 min followed by reperfusion for different time periods. Sham, sham operated control group; I/R, ischemia (30 min)-reperfusion group. (D) Fendrr expression was detected using qRT-PCR after 30 min of ischemia followed by reperfusion for different time periods (0, 2, 6, 12, and 24 h) in 8–12 weeks old Balb/c mice. *, *P*<0.05, vs. sham group, n = 3; **, *P*<0.01, vs. sham group, n = 3. (E) The localization of Fendrr was determined using FISH assay with 18s rRNA as internal control in cardiomyocytes. (F) Fendrr expression was detected using qRT-PCR in cardiomyocytes treated with different H_2_O_2_ concentrations (0, 0.1, 0.25, and 0.4 mM). ***, *P*<0.001, vs. 0 mM group, n = 4. (G) Fendrr expression was detected using qRT-PCR in cardiomyocytes treated with H_2_O_2_ (0.4 mM) for different time periods (0, 6, 12, and 24 h). ***, *P*<0.001, vs. 0 mM group, n = 4; ****, *P *< 0.0001, vs. 0 h group, n = 4.

To investigate the expression of Fendrr in myocardial injury, we established a mouse model of MI/R injury and observed changes in electrocardiograms (Figure 2A), serum creatine kinase (Figure 2B) levels, and hematoxylin–eosin staining (Figure 2C), which suggested that the mouse model of MI/R injury was successful. We then detected Fendrr expression in the injured mouse heart tissues at different time points using qRT-PCR. The results showed that Fendrr expression level decreased to the lowest level after 6 h but gradually increased at 12 h and approached the basal level at 24 h of reperfusion (Figure 2D). We used RNA FISH to determine the degree of Fendrr localization in cardiomyocytes and confirmed high Fendrr enrichment in the cytoplasm of H9c2 cells (Figure 2E). H_2_O_2_ treatment of H9c2 cells simulated cardiomyocyte injury *in vitro.* We found that Fendrr expression significantly decreased in the H_2_O_2_-induced cardiomyocyte injury model with increasing H_2_O_2_ concentrations (Figure 2F), whereas its expression gradually decreased as the duration of H_2_O_2_ treatment increased (Figure 2G). Thus, Fendrr was downregulated in MI/R injury and cardiomyocyte injury induced by oxidative stress.

### LncRNA Fendrr relieved H2O2-induced cardiomyocyte injury

**Figure 3.**
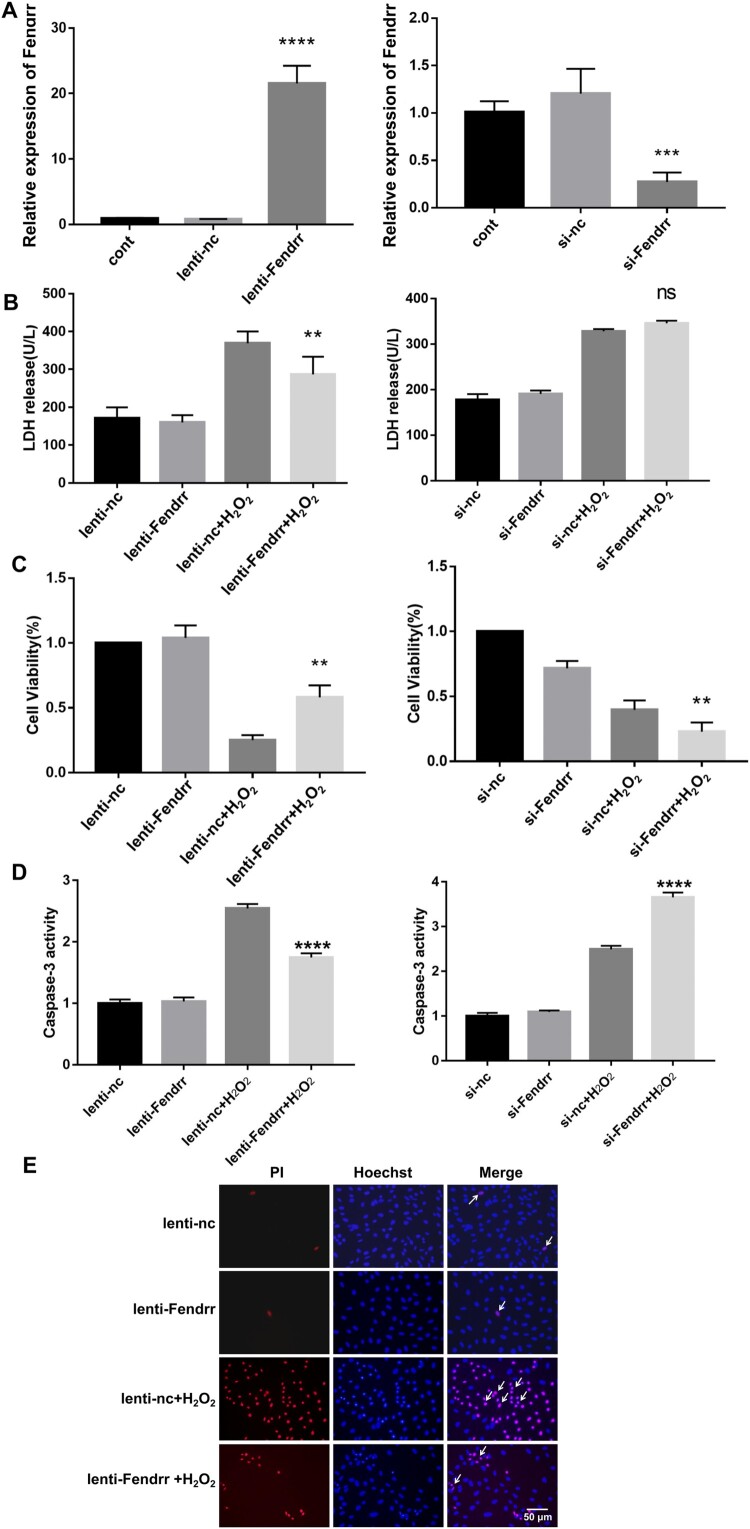
LncRNA Fendrr relieved H_2_O_2_-induced cardiomyocyte injury. (A) The overexpression or knockdown efficiency of Fendrr were measured in H9c2 cells after transfection with Fendrr overexpression vector or siRNAs. Fendrr expression was not affected by transfection. All experiments were repeated at least thrice Data are represented as mean ± SD. ***, *P* < 0.001; ns, not significantly. (B) Effect of Fendrr overexpression or knockdown on lactate dehydrogenase (LDH) release in cardiomyocytes with H_2_O_2_ (0.4 mM, 12 h) treatment. ns, not significantly, **, *P* < 0.001. (C) The effect of Fendrr overexpression or knockdown on cell viability in cardiomyocytes with H_2_O_2_ (0.4 mM, 12 h) treatment. **, *P *< 0.01, vs. lenti-nc + H_2_O_2_ group, n = 4; **, *P* < 0.001. (D) The caspase-3 activity was evaluated in cardiomyocytes with Fendrr overexpression or knockdown with H_2_O_2_ (0.4 mM, 12 h) treatment. si-nc, negative control; si-Fendrr, Fendrr siRNA. lenti-nc, the empty lentivirus vector served as negative control; lenti-Fendrr, the lentivirus vector containing Fendrr overexpression plasmid. Data are represented as mean ± SD. ***, *P* < 0.001. (E) Propidium iodide staining was performed in lentivirus-transfected cardiomyocytes with PBS or H_2_O_2_ (0.4 mM, 12 h) treatment. The cells indicated by the arrow are representative of apoptotic cells. lenti-nc, the lentivirus empty vector served as negative control; lenti-Fendrr, the lentivirus vector containing Fendrr overexpression plasmid.

To explore the role of Fendrr in H_2_O_2_-induced cardiomyocyte injury, we knocked down its expression using small interfering RNA (siRNA) and overexpressed it in H9c2 cardiomyocytes (Figure 3A). LDH was released from cardiomyocytes and the upregulation of Fendrr reduced LDH release from injured cardiomyocytes after H_2_O_2_ treatment (Figure 3B). Evaluating the cell viability post H_2_O_2_-induced cardiomyocyte injury, we found that Fendrr overexpression alleviated the decrease in cell viability induced by H_2_O_2_; however, cell viability further decreased after Fendrr knockdown (Figure 3C). Caspase-3 activity was detected in cardiomyocytes, and the data showed that Fendrr overexpression alleviated the cell apoptosis induced by H_2_O_2_; however, cell apoptosis further increased after reducing Fendrr (Figure 3D). Propidium iodide staining indicated apoptosis by staining the nuclei of H_2_O_2_-treated cardiomyocytes red, indicating cell damage. Fendrr upregulation significantly reversed H_2_O_2_-induced damage in cardiomyocytes compared to that in H_2_O_2_-treated cells (Figure 3E). Thus, Fendrr protects cardiomyocytes from H_2_O_2_-induced injury.

### Nucleolin up-regulated the expression of LncRNA Fendrr in H2O2-induced cardiomyocytes injury

**Figure 4.**
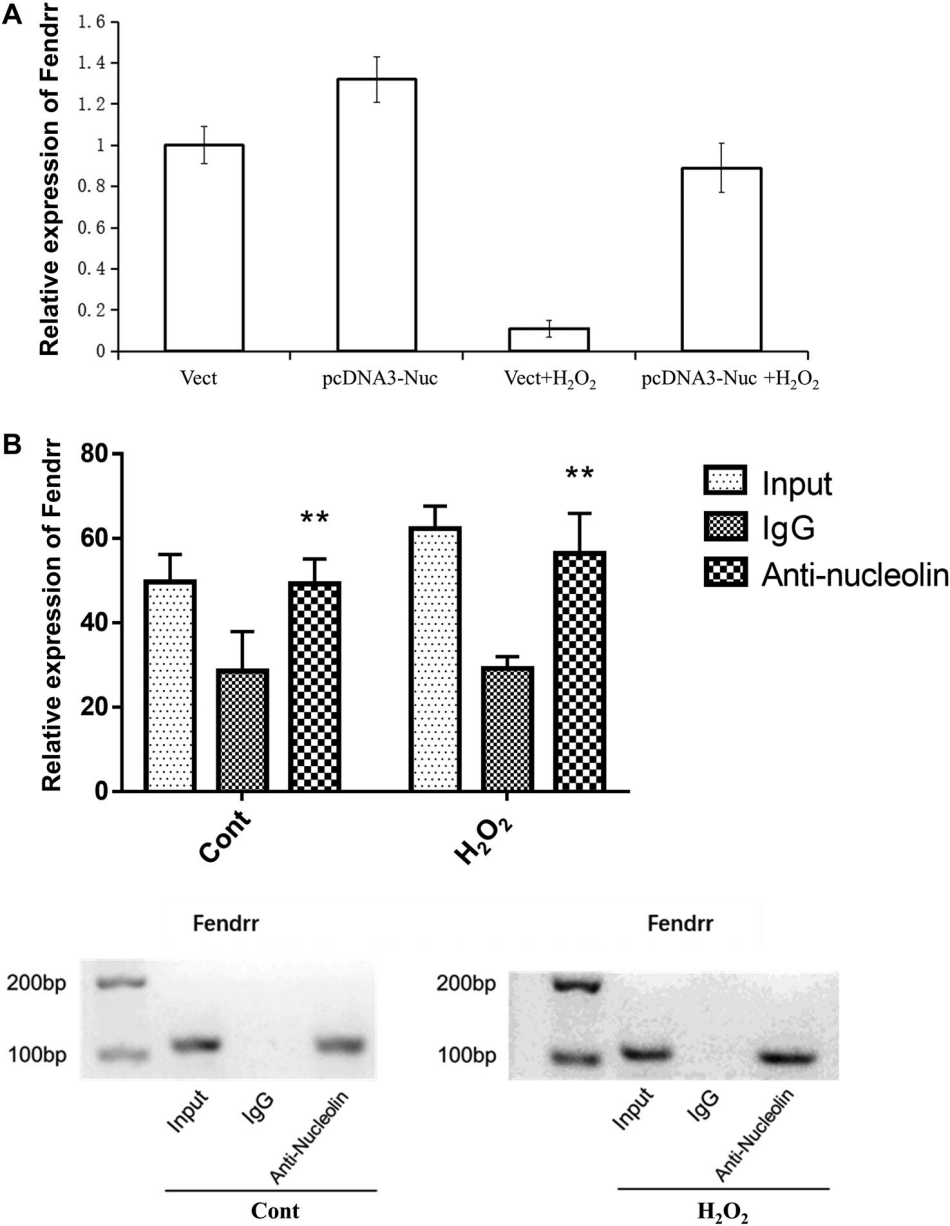
Nucleolin up-regulated the expression of LncRNA Fendrr in H_2_O_2_-induced cardiomyocytes injury. (A) Fendrr expression was detected using qRT-PCR. Cardiac muscle cell line with nucleolin overexpression was treated with H_2_O_2_ for 12 h. *, *P* < 0.01, vs. Vect group, n = 6; #, *P *< 0.01, vs. Vect + H_2_O_2_ group, n = 6. (B) The interaction between nucleolin and Fendrr was confirmed using RNA immunoprecipitation (RIP) (top) and electrophoresis on agarose gel (bottom) in cardiomyocytes treated with PBS or H_2_O_2_ (0.4 mM, 12 h). **, *P* < 0.01, vs. IgG group, n = 4. Vect, the empty vector served as negative control; pcDNA3.1-Nuc, nucleolin overexpression group.

To investigate the effect of nucleolin on Fendrr expression in cardiomyocytes, H9c2 cells overexpressing nucleolin were treated with H_2_O_2_ for 12 h, and nucleolin overexpression increased the expression of Fendrr in H_2_O_2_-induced cardiomyocyte injury (Figure 4A). RIP results also revealed that nucleolin could bind to Fendrr (Figure 4B). Thus, nucleolin regulates Fendrr expression by interacting with Fendrr in cardiomyocytes.

### Nucleolin protects H2O2-induced injury by up-regulating Fendrr in cardiomyocytes

**Figure 5.**
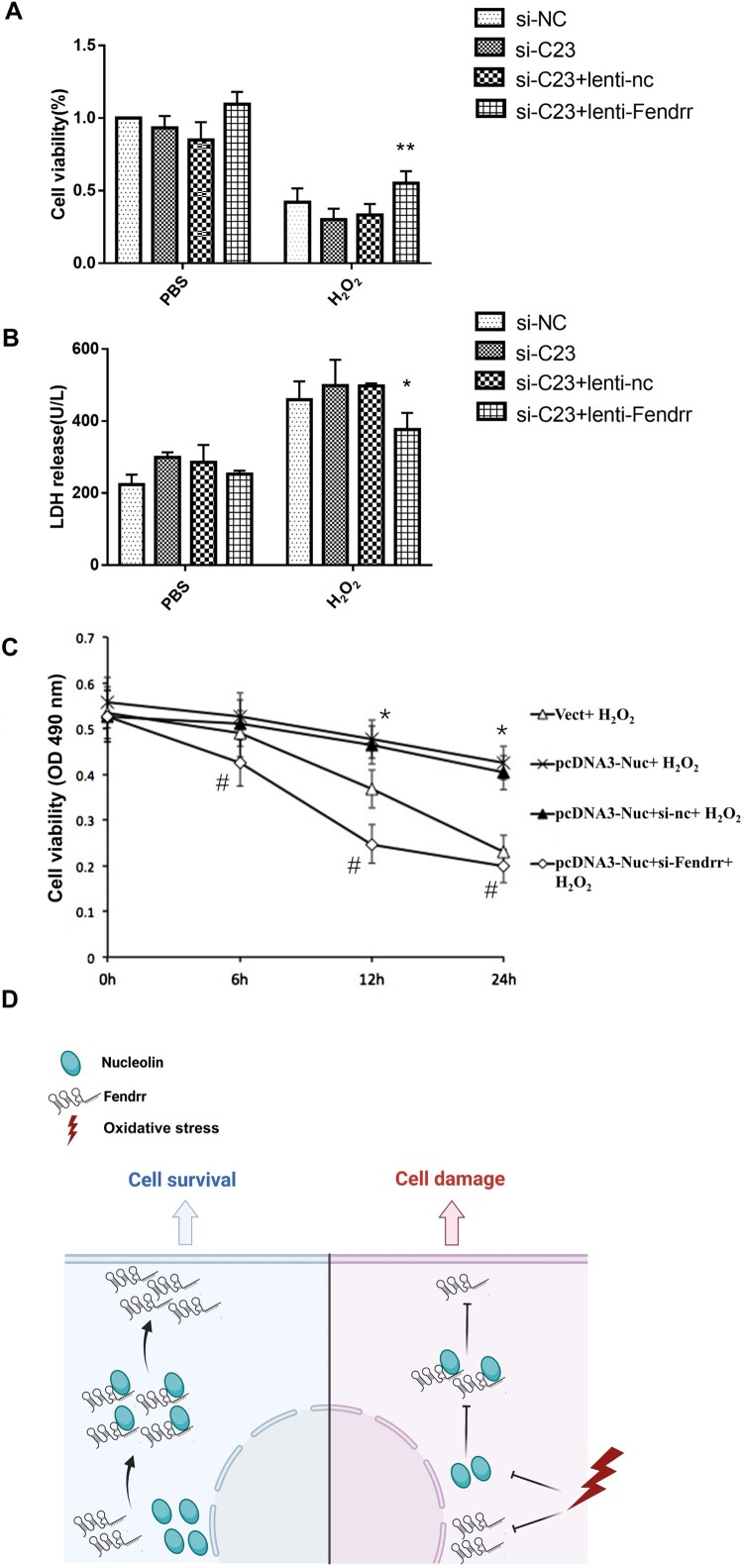
Nucleolin protected H_2_O_2_-induced injury by up-regulating lncRNA Fendrr in cardiomyocytes. (A) The effect of Fendrr overexpression on the decrease of cell viability mediated by nucleolin ablation and H_2_O_2_ exposure. Cell viability was detected in siRNA- or lentivirus-transfected cardiomyocytes with PBS or H_2_O_2_ (0.4 mM, 12 h) treatment. **, *P *< 0.01, vs. si-C23 + lenti-nc group, n = 4; (B) The effect of Fendrr overexpression on the LDH release induced by nucleolin ablation and H_2_O_2_ exposure. The LDH release level in culture medium was tested. *, *P *< 0.05, vs. si-C23 + lenti-nc group, n = 3; (C) The effect of Fendrr knockdown on the protective effect exerted by nucleolin in cardiomyocytes. *, *P *< 0.01, vs. Vect + H_2_O_2_ group, n = 6. #, *P *< 0.01, vs. pcDNA3-Nuc + Scramble + H_2_O_2_ group, n = 6. si-NC, negative control; si-C23, nucleolin siRNA; lenti-nc, the empty lentivirus vector served as negative control; lenti-Fendrr, the lentivirus vector containing Fendrr overexpression plasmid. Vect, the empty vector served as negative control; pcDNA3-Nuc, overexpression nucleolin group; si-nc, negative control; si-Fendrr, Fendrr siRNA. (D) Diagram summarizing the inferences: Fendrr is down-regulated in cardiomyocytes after H_2_O_2_ exposure, nucleolin interacts with Fendrr and up-regulates Fendrr expression, and nucleolin protects myocardial cells against oxidative stress by up-regulating Fendrr expression. Paragraph: use this for the first paragraph in a section, or to continue after an extract.

To further explore the effect of Fendrr on the cardioprotective role of nucleolin during oxidative stress, we co-transfected the cells with Fendrr lentiviral vector and nucleolin siRNA. The results indicated that low nucleolin expression could further promote the decline of cardiomyocyte viability caused by H_2_O_2_, but Fendrr overexpression in cells with nucleolin knockdown showed significantly improved cell viability after H_2_O_2_ treatment (Figure 5A). Similarly, we measured LDH levels in the cell culture medium and found that Fendrr overexpression partially inhibited LDH release from H_2_O_2_-treated cells (Figure 5B). Furthermore, we co-transfected Fendrr siRNA and a nucleolin vector into H9c2 cells, and low Fendrr expression eliminated the protective effect of nucleolin against H_2_O_2_-induced cardiomyocyte injury (Figure 5C). Thus, nucleolin could interact with Fendrr, upregulate Fendrr expression, and inhibit H_2_O_2_-induced cell apoptosis in cardiomyocytes (Figure 5D). Fendrr is involved in the protective effects of nucleolin in cardiomyocytes during oxidative stress.

## Discussion

LncRNAs play numerous roles in biological processes. Many studies have investigated the intracellular mechanisms of functional lncRNAs, which can bind to multiple molecules [18, 19]. lncRNA Fendrr was discovered for the first time during its interaction with polycomb inhibitory complex 2 (PRC2) during mouse heart development [20]. According to bioinformatics analysis and RIP experiment results, Fendrr may bind to the PRC2 complex and the MLL histone modification complex to play a functional role [21–23]. Fendrr enhances doxorubicin resistance in doxorubicin-resistant and-sensitive human osteosarcoma cells by negatively regulating the post-transcriptional expression of the multidrug resistance-related proteins ABCB1 and ABCC1 [24]. Fendrr transcription levels are negatively correlated with SOX4 protein levels in colon cancer; SOX4 protein is one of the binding targets of Fendrr, and Fendrr-mediated SOX4 inhibition may be essential in reducing colon cancer progression [25]. However, the role of Fendrr in human cardiovascular disease remains unclear. The negative association of Fendrr levels in peripheral blood mononuclear cells with the left ventricular mass index in hypertensive individuals revealed that it may play a cardioprotective role [26]. In addition, Fendrr polymorphism was recently found to be significantly associated with the risk of developing hypertrophic cardiomyopathy (HCM) using NGS. This study found that rare alleles in the Fendrr polymorphism were significantly protective against HCM [27]. lncRNA Fendrr was significantly upregulated in a mouse model of MI/R injury and could interact with nucleolin in cardiomyocytes. Moreover, overexpression of lncRNA Fendrr plays a protective role against H_2_O_2_-induced injury in cardiomyocytes. Also, Fendrr is involved in the myocardial protective role of nucleolin during oxidative stress. Therefore, lncRNA Fendrr could be a new biomarker for the early diagnosis, therapeutic target, and prognosis prediction of heart diseases.

LncRNA-protein interactions primarily reveal the post-transcriptional regulatory mechanism. So far, research has revealed related molecular mechanisms of the interaction between long noncoding RNA and nucleolin, which could lead to a plethora of useful targets for disease diagnosis and prevention. For example, lncRNA CYP4B1-PS1-001 may influence nucleolin ubiquitination and degradation, making it a promising target for diabetic nephropathy [15]. The lncRNA CYTOR is significantly upregulated in colon cancer tissue samples and is associated with poor prognosis. The complex formed by the CYTOR-nucleolin interaction activates signaling pathways that promote colon cancer progression [28]. Our inference that Fendrr is important for the protective role of nucleolin implies that nucleolin may exert a protective function by interacting with Fendrr during cardiomyocyte injury and provides a new perspective on the mechanism through which nucleolin protects against myocardial injury.

The function of nucleolin depends mainly on the RNA-binding domain in the central region [29]. Nucleolin binds to the G-rich regions of mRNA and lncRNAs. Also, nucleolin combines with the G-rich regions of SNHG1, which partially indicates the nuclear retention of SNHG1 in disease states [30]. Additionally, nucleolin directly binds to lncRNA Dnm3os and enhances Dnm3os-induced inflammation-related gene expression [31]. Nucleolin can also protect cardiomyocytes from DOX-induced damage by interacting with miR-21 and upregulating miR-21 expression [32]. Our results suggest that nucleolin may play a protective role in myocardial injury by binding to Fendrr and regulating its expression. Thus, exploring the answers for questions such as what is the specific mechanism underlying nucleolin regulation of Fendrr in cardiomyocytes and other molecules mechanism would provide researchers with an in-depth knowledge of the pathogenesis of myocardial injury and target identification for its prevention and treatment. These questions will be our future studies direction, and other researchers are welcome to explore with us.

## Conclusions

In conclusion, we discovered that lncRNA Fendrr plays an important role in the cardioprotective effect of nucleolin against H_2_O_2_-induced injury. lncRNA Fendrr may be a potential therapeutic target for myocardial injury during oxidative stress.

## Data Availability

The data used and analyzed during the current study are available from the corresponding author upon reasonable request.

## Abbreviations

**Abbreviations**: MI/R: myocardial ischemia-reperfusion; H_2_O_2_: hydrogen peroxide; lncRNA: long noncoding RNA; Fendrr: FOXF1 adjacent noncoding developmental regulatory RNA; LDH: lactate dehydrogenase; PI: Propidium iodide; AMI: acute myocardial infarction; CCK-8: cell counting Kit-8; qRT-PCR: quantitative real-time polymerase chain reaction; RIP: RNA immunoprecipitation; SD: standard deviation; siRNA: small interfering RNA; NCL: Nucleolin; HCM: hypertrophic cardiomyopathy.
